# TADF and exciplex emission in a xanthone–carbazole derivative and tuning of its electroluminescence with applied voltage†

**DOI:** 10.1039/c9ra08227a

**Published:** 2019-12-04

**Authors:** Qamar T. Siddiqui, Ankur A. Awasthi, Prabhjyot Bhui, Pradnya Parab, Mohammad Muneer, Sangita Bose, Neeraj Agarwal

**Affiliations:** School of Chemical Sciences, UM-DAE, Centre for Excellence in Basic Sciences, University of Mumbai Santacruz (E) Mumbai 400098 India na@cbs.ac.in; School of Physical Sciences, UM-DAE, Centre for Excellence in Basic Sciences, University of Mumbai Santacruz (E) Mumbai 400098 India sangita@cbs.ac.in; Department of Chemistry, Aligarh Muslim University Aligarh India

## Abstract

Materials showing white light emission have found applications in a variety of solid state devices especially in display technology. For white light emission, doping of red (R), green (G) and blue (B) emitters in a host matrix is commonly practised. However, finding RGB emitters of similar stability with homogenous doping is challenging. Furthermore, such devices suffer from color purity in the long run. Small organic light emitters, capable of colour tuning and having a broad emission spectrum are in high demand as they provide colour stability, reproducibility, a simple device geometry and high efficiency. Recently, it has been shown that the efficiency of OLEDs can be enhanced by employing thermally activated delayed fluorescence (TADF) materials. Here, we designed and synthesised a xanthone–carbazole based D-A-D material (Xan-Cbz) for TADF properties. Blue TADF emission, in neat thin films, at 470 nm was observed and further investigated by studying delayed fluorescence and lifetime measurements. In addition, a blend of Xan-Cbz with NPD shows exciplex emission at 525 nm in thin film. OLEDs based on Xan-Cbz were fabricated using several device configurations. OLEDs having the device configuration ITO/PEDOT:PSS/NPD/Xan-Cbz/Bphen/LiF-Al showed a luminance of 1.96 × 10^4^ Cd m^−2^ (at a current density of 50 mA cm^−2^) and *V*_ON_ at ∼6 V. Electroluminescence showed the features of both neat emission (470 nm) of Xan-Cbz and its exciplex (525 nm) with NPD. Further, colour tuning was observed as a function of applied voltage and the ratio of light intensity (*I*_525_/*I*_470_) of neat and exciplex emission was found to decrease with increasing voltage. Greenish-blue emission (CIE coordinates: 0.202, 0.382) from Xan-Cbz OLEDs was obtained. Xan-Cbz showed its neat emission (at 470 nm) in ITO/PEDOT:PSS/CBP/Xan-Cbz/Bphen/LiF-Al and pure exciplex emission (at 525 nm) in ITO/PEDOT:PSS/NPD:Xan-Cbz/Bphen/LiF-Al device configurations. Thus in this article we showed blue TADF emission, exciplex emission and voltage dependent color tuning in OLEDs based on a small organic emitter.

## Abstract 2

Xanthone–carbazole (Xan–Cbz) derivative is synthesized and its photophysical properties are explored. OLEDs of Xan–Cbz shows tunability of electro-luminescence with applied voltage.

## Graphical abstract

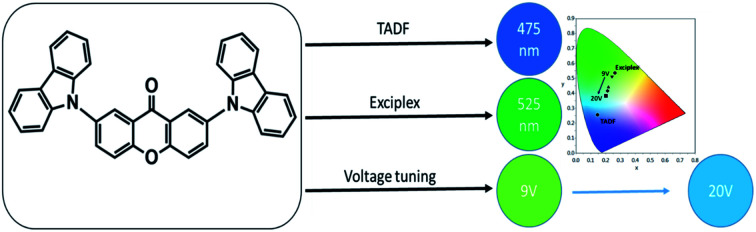

## Introduction

**Fig. 1:**
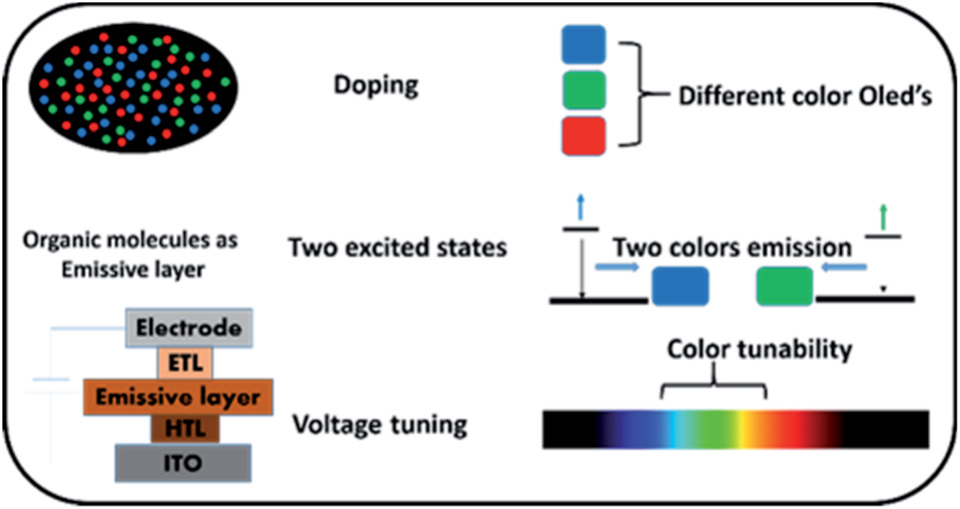
Various strategies for multi-coloured emission from OLEDs.

Organic light emitting devices (OLEDs) are popular for active-luminescent displays and lighting technologies, owing to their special features, such as easy processing, light weight, flexibility, commercial viability *etc.*^1–12^ For white light, emission of primary colours (RGB) or a broad visible-light spectrum is vital. Several strategies have been reported by various groups to obtain white light (Fig. 1). Commonly, premeditated mixing of blue, green and red emitters or blue and orange emitters has been applied in devices to obtain white light OLEDs.^13–20^ In such devices, emitters of divergent colours are doped in a suitable host matrix especially when phosphorescent materials are in action. Use of several emitters and hosts make the device architecture very complex thus causing tedious device fabrication. Tandem OLEDs have been considered for multi coloured emission by using polymer as well as small organic molecules.^21,22^ Such OLEDs provide easy accessibility of broad/white light emission; however, the device geometry becomes complicated as an additional electrode is required. In few devices reported in the literature, processes like exciplex formation and Auger recombination has been shown leading to variable colour emission depending on the applied voltage.^23,24^

To simplify the device fabrication process, single organic light emitters which are capable of colour tuning and broad emission spectrum are essential. Single emitter with tunability in OLED is advantageous as it provides colour stability, reproducibility, and simple device geometry. To facilitate this, new molecules have to be designed which are capable of producing emission in wide visible region. More than one emission from single molecule can be achieved by having radiative decay from two different excited states or formation of excimer and exciplex emission in solid state. In order to improve the efficiency of OLEDs, involvement of singlet and triplet excited states is advantageous. This can be achieved by employing the phosphorescent emitters, with molecules having thermally activated delayed fluorescence (TADF) or triplet–triplet annihilation (TTA) features.^25–32^ We recently reported TADF in undoped acridone–carbazole derivatives and their exciplex emission in OLEDs.^33^ Generally, exciplex is studied in the blend of suitable donor and acceptor molecules to achieve new emission different than that of individual donor and acceptor materials. In devices, when hole transporting materials capable of forming exciplex with emitter is placed beside it, formation of exciplex at the interface of two layers can be expected. In the same device emission from neat emitter can also be obtained when recombination of excitons happens in the emissive layer. Exciton recombination depends on several factors *e.g.* alignment of energy levels of different materials used in the device, charge carrier mobilities, thickness of layers, applied voltage *etc.* Suitable device geometry can help in obtaining the emission from neat emitter and exciplex simultaneously. Here, in this manuscript, we report on the design and synthesis of xanthone–carbazole (Xan-Cbz) derivative which shows blue TADF (∼470 nm) in undoped film and an exciplex emission (∼525 nm) when blended with NPD (*N*,*N*′-di(1-naphthyl)-*N*,*N*′-diphenyl-(1,1′-biphenyl)-4,4′-diamine). OLEDs of Xan-Cbz were fabricated in different device geometries to ascertain which of the two emissions (∼470 or ∼525 nm) is more dominant in the device. It was observed that for the device architecture where NPD was used as a hole transporting layer prior to the active layer, exciplex emission at 525 nm was obtained from the interface, accompanied by TADF emission from the active layer of Xan-Cbz. Interestingly, for such devices, the ratio of light from the neat emitter (Xan-Cbz) and its exciplex was seen to be tunable with the applied voltage. Thus, our results show that Xan-Cbz can be used as a green or a blue emitter depending on the bias voltage in the OLED device.

## Results and discussion

Recently, we have reported palladium free amination of acridone with carbazole using 1,10-phenanthroline and copper iodide as catalysing agents.^32^ Xan-Cbz was synthesized in a similar way (see Scheme S1 of the ESI†). In this reaction, mixture of mono- and di amines were formed along with unreacted carbazole. In column chromatography, carbazole and Xan-Cbz moved together as they have identical retention factor (Rf). For further purification chemical method was used (see ESI†) followed by column chromatography and recrystallization. Characterization of Xan-Cbz was carried out by several spectroscopic techniques *e.g.*^1^H-NMR, ^13^C-NMR, MALDI-TOF (see ESI†).

**Fig. 2:**
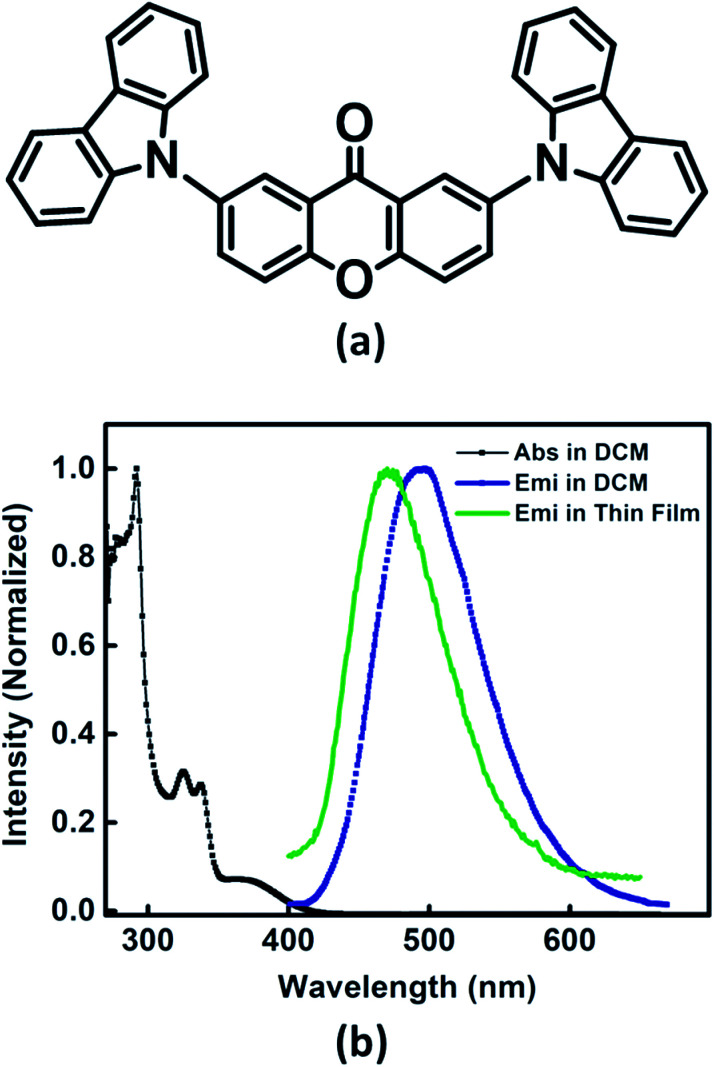
(a) Molecular structure of Xan-Cbz and (b) absorption, emission spectra in DCM and emission spectrum in thin film.

Ground and excited state photophysical properties of Xan-Cbz was studied by using absorption and emission spectroscopies in both solution and thin films. Absorption and emission spectra of Xan-Cbz, in dichloromethane and thin films, were recorded and are shown in Fig. 2. Photophysical data is summarized in Table 1. In absorption spectra, a low intensity broad peak was observed at 370 nm for Xan-Cbz. Two high intensity peaks at 265–310 nm were also observed. The broad, low intensity peak is attributed to charge transfer band and further explored by solvatochromic studies. A slight shift is observed in absorption spectra in various polarity solvents. Contrary to absorption spectra, there is a significant red shift of ∼90 nm between low polarity (Toluene, 470 nm) and high polarity (DMSO, 560 nm) solvents in the emission spectra. It is well known that the emission spectra arising from locally exited (LE) S_1_–S_0_ does not show significant shift with increasing polarity whereas the emission spectra arising from charge transfer (CT) states are broad and shows dependence on polarity of the solvent medium.^34–38^ Solvent dependent studies of Xan-Cbz suggest that the emission maxima is arising from ^1^CT transition. The emission maxima for Xan-Cbz in dichloromethane was found to be at 493 nm and a hypsochromic shift of ∼20 nm was observed in thin films (470 nm).

**Table 1:** Photophysical and electrochemical data of Xan-Cbz

*λ*_abs_ a nm (log *ε*)	*λ*_abs_ b (nm)	*λ*_em_ a (nm)	*λ*_em_ b (nm)	*E*_HOMO_ c (eV)	*E*_LUMO_ d (eV)	*ϕ*	*τ*_PF_ (μs)	*τ*_TADF_ (μs)	*E*_S1_ (eV)	*E*_T1_ (eV)	Δ*E*_ST_ (eV)
292 (4.13), 325 (4.14), 338 (4.09), 370 (3.48)	387	493	470	6.0	3.0	22	0.011 (in air), 0.016 (in N2)	3.8	2.42	2.10	0.32

a In dichloromethane.

b In thin film.

c *E*_HOMO_ = −(*E*_[ox *vs.* Fc/Fc^+^]_ + 5.1) eV.

d *E*_LUMO_ = −(*E*_[red *vs.* Fc/Fc^+^]_ + 5.1) eV.

For the TADF prospects, the difference in the singlet and triplet energy levels (Δ*E*_ST_) is required to be estimated. It is known that triplet states quenches at room temperature, therefore, phosphorescence emission was recorded at 77 K in 2-methyltetrahydrofuran (2-MeTHF). To populate the triplet state ethyl iodide (10% v/v) was added to the solvent as it sensitizes the triplet state by heavy atom effect. Emission spectra of Xan-Cbz at low temperature in presence of ethyl iodide is shown in ESI.† Xan-Cbz showed two peaks at 510 and 590 nm. Peak at ∼510 nm is assigned to fluorescence emission and the new peak at ∼590 nm is accounted for phosphorescence. Singlet and triplet energy levels were estimated from the peak maxima of fluorescence and phosphorescence peaks respectively. Singlet and triplet energy levels of Xan-Cbz are summarized in Table 1. From *E*_S1_ and *E*_T1_, Δ*E*_ST_ was found to be 0.32 eV. Energy gap between singlet and triplet energy levels is similar to those of TADF materials recently used in OLEDs.^39–41^ Thus, we believe that these materials can find applications in OLEDs.

In order to use Xan-Cbz in solid state devices it is important to find out the highest occupied molecular orbital (*E*_HOMO_) and lowest unoccupied molecular orbital (*E*_LUMO_) energy levels. The *E*_HOMO_ and *E*_LUMO_ were estimated using the cyclic voltammetry measurements and optical band gap. For organics, first oxidation potential can be considered as its HOMO energy level. The first oxidation potential (*E*_ox_) of Xan-Cbz with respect to oxidation potential of ferrocene (Fc/Fc^+^, as internal standard) was found out. *E*_ox_ was observed at 0.9 V higher than that of Fc/Fc^+^ (see ESI†). Using *E*_HOMO_ = *E*_ox_ w.r.t Fc/Fc^+^ + 5.1 eV,^42–44^ HOMO energy level was estimated to be −6.0 eV. The optical gap, as measured from the intersection of absorption and emission bands of Xan-Cbz is ∼3.0 eV. Using *E*_HOMO_ and optical gap, *E*_LUMO_ were estimated at ∼−3.0 eV.

**Fig. 3:**
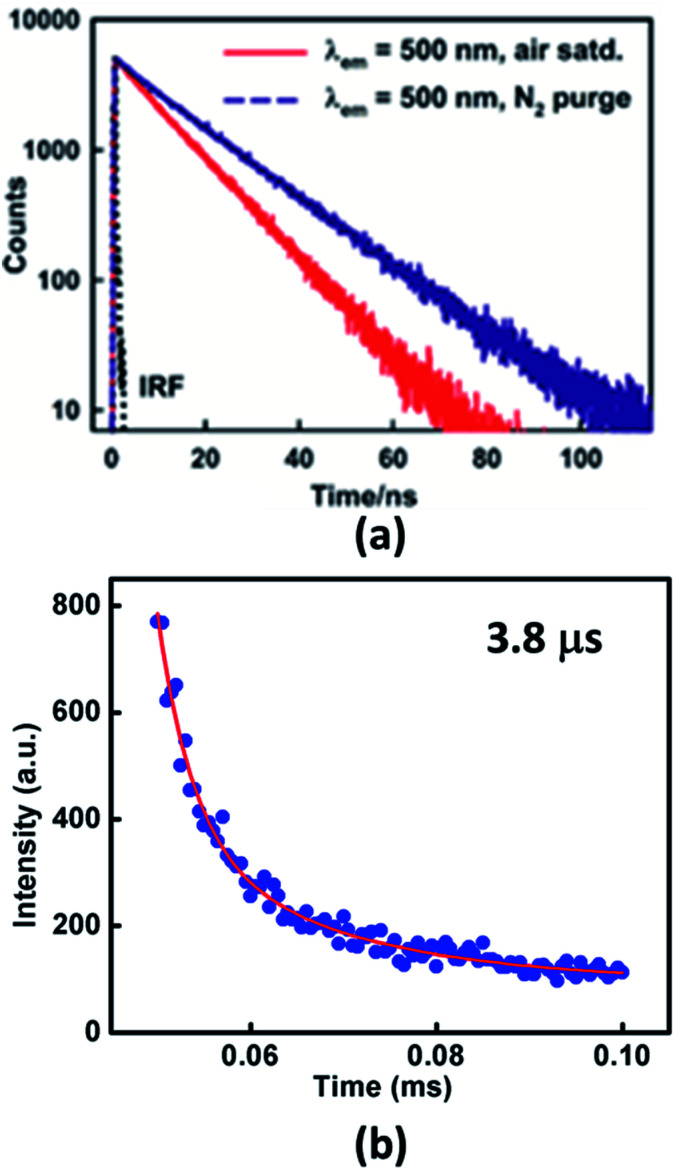
Fluorescence decay profiles of Xan-Cbz in (a) air saturated and nitrogen purged THF (b) in thin film.

To establish the TADF property in Xan-Cbz fluorescence decay was studied in several experimental conditions. Transient measurement at room temperature showed single exponential decay arising from prompt fluorescence (Fig. 3). Fluorescence life times observed for Xan-Cbz is ∼11 ns. Difference between singlet and triplet energy levels of Xan-Cbz is small, therefore, triplet state may also contribute to emission lifetime as it happens in TADF materials. To get an idea about the contribution of triplet state, life time studies were also carried out under nitrogen purged solvent as it eliminates the triplet quenching due to the presence of oxygen and therefore enhances the utilization of triplet states in emission. Life times for Xan-Cbz in nitrogen purged solvent increased by ∼50% and observed to be ∼16 ns (Fig. 3). These observations further suggest the close proximity of singlet and triplet energy levels.^40,45^

The life time of triplet state is generally in microsecond–millisecond (μs–ms) range. Long life time of triplet states may results in triplet–triplet annihilation which is not as efficient as TADF.^46–51^ Hence, it is desirous to have short triplet life time to have better TADF which should also results in short TADF life time. We determined the TADF life time of Xan-Cbz in undoped thin films. The fluorescence decay profile (at 470 nm) were recorded after a delay of 50 μs. TADF life time for Xan-Cbz was estimated to be 3.8 μs. As mentioned earlier, the prompt fluorescence life time is much less than 100 ns (Table 1), hence, spectra obtained after a delay of 50 μs rules out the possibility of any component due to prompt fluorescence. Thus, emission decay obtained after a delay is attributed to TADF.

**Fig. 4:**
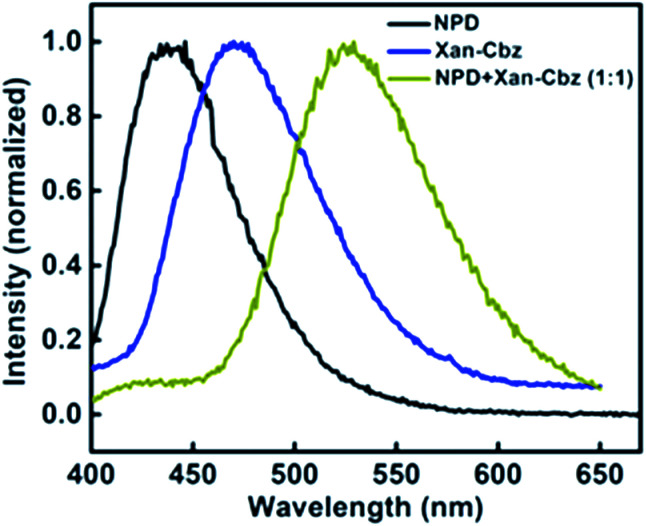
Emission spectra of Xan-Cbz, NPD and its blend (1 : 1) in thin films.

In blends of electron donor and acceptor, excited state formation by electron transfer from donor to acceptor can be envisaged. Such excited states are, generally, composed of excited state of donor and ground state of acceptor molecule and are called as exciplex. Exciplex shows different emission from both of its components *i.e.* it does not match with the individual emission of donor and/or acceptor. Also, exciplex emission is found to be broader and structure less. It is important to have suitable HOMO and LUMO energy levels of donor and acceptor as they play an important role in charge transfer and thus in exciplex formation. It has been suggested that HOMO and LUMO of donor should be more than that of acceptor. For efficient charge transfer the energy difference between the LUMOs of donor and acceptor should be at least 0.5 eV as lesser difference will cause energy transfer. We studied the exciplex formation in blend of Xan-Cbz with NPD and CBP (4,4′-bis-(*N*-carbazolyl)-1,1′-biphenyl). In the blend with NPD, NPD acts as electron donor while Xan-Cbz as acceptor. Emission of blend of NPD with Xan-Cbz is shown in Fig. 4. A new emission peak is observed with maxima at 525 nm which does not belong to either NPD or Xan-Cbz. This new peak is assigned to exciplex emission. Fluorescence lifetime of this new emission is also recorded (see Fig. S7†). Long life time is an indication of involvement of TADF in exciplex formation. No exciplex formation was observed for the blend of Xan-Cbz with CBP as HOMO and LUMO energy levels of CBP are not compatible for the exciplex formation with Xan-Cbz.

**Fig. 5:**
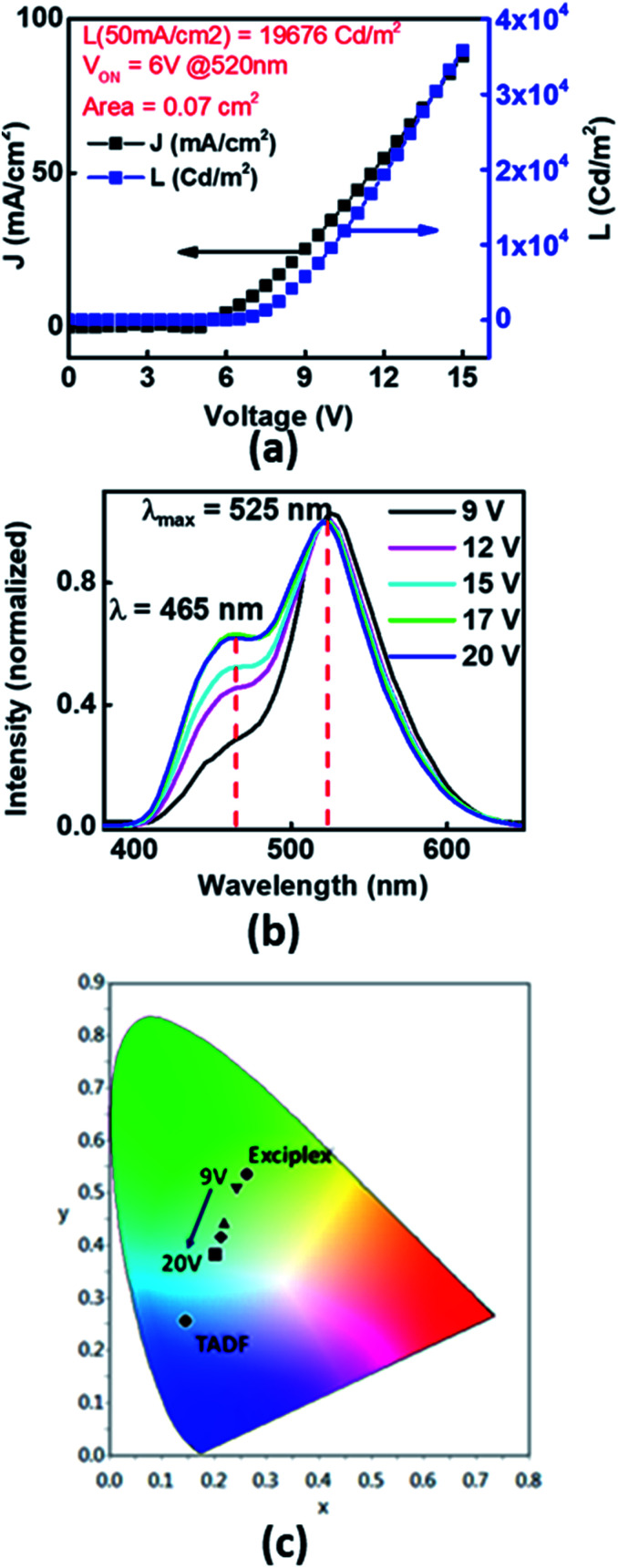
(a) Device, ITO/PEDOT:PSS/NPD/Xan-Cbz/Bphen/LiF-Al, characteristics (*J* and *L vs.* bias voltage) of Xan-Cbz; (b) normalized electroluminescence of the device at different voltages and (c) color gamut showing the emission color at different voltages.

To study the prospects of Xan-Cbz in OLEDs, devices in different geometries were fabricated and studied. One of the device geometry which was studied was ITO/PEDOT:PSS/NPD/Xan-Cbz/Bphen/LiF-Al. In this device, poly(2,3-dihydrothieno-1,4-dioxin)-poly(styrenesulfonate) (PEDOT:PSS) was used as a HTL. Since, *E*_HOMO_ of NPD is very close to *E*_HOMO_ of Xan-Cbz, a thin layer of NPD (∼40 nm) was used in between the PEDOT:PSS and Xan-Cbz which was expected to further facilitate the hole transport. Bathophenanthroline (Bphen) was used as a hole blocking layer (HBL). In these devices, while PEDOT:PSS was spin coated with the usual protocol to give ∼50 nm films, the rest of the layers were thermally evaporated under vacuum (with a base vacuum of 1 × 10^−6^ mbar). The active area of the devices were in the range of 0.05–0.08 cm^2^. Turn on voltage, *V*_ON_ for this geometry was found to be ∼6 V. Bright light was obtained showing a luminance as high as 1.96 × 10^4^ Cd m^−2^ (@ a current density of 50 mA cm^−2^) as can be seen from the device characteristics shown in Fig. 5(a). Furthermore, an interesting phenomenon was observed in these devices. At biases of 6–12 V, the colour of the emission from the devices visually appeared predominantly green. However, at higher biases (12–20 V), the devices appeared bluish green. The electroluminescence spectra at different voltages is shown in Fig. 5(b). Here, the EL spectra are shown for different bias voltages which are normalized with the light intensity recorded at 9 V at 525 nm. Two important points can be identified from the EL spectra. (i) At all voltages two peaks are seen at ∼465 nm which matches with the emission of Xan-Cbz in thin films (see Fig. 4) and at ∼525 nm which matches with the PL emission (exciplex) of blend of Xan-Cbz and NPD (see Fig. 4). (ii) The ratio of the light intensity at 525 and 465 nm, is seen to decrease with increasing bias voltage which implies a voltage tunability of the light emission in these devices. In these devices, the exciplex is formed only at the narrow interface of NPD and Xan-Cbz. At lower voltages, exciplex emission is dominant, which can be explained by assuming that lesser number of carriers transported to the active layer (Xan-Cbz) and hence the exciplex formation at the interface is facilitated. As expected, with increase of applied voltage, more carriers will reach the active layer of Xan-Cbz. Thus, exciton recombination occurs in the active layer of Xan-Cbz as well as at the interface of Xan-Cbz and NPD at higher voltages. Emission from neat Xan-Cbz and its exciplex with NPD (at interface) leads to broader spectral range of light from OLED. Colour tuning of device with applied voltage is clearly visible from the colour gamut showing the CIE coordinates of the emission (Fig. 5(c)).

**Fig. 6:**
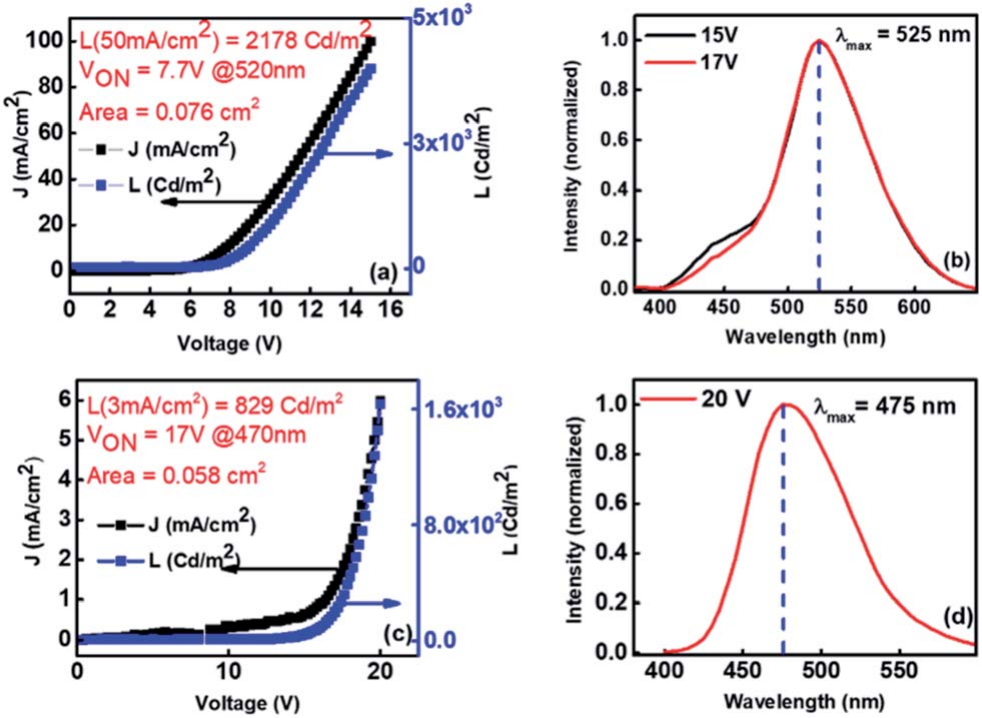
(a) Device, ITO/PEDOT:PSS/NPD:Xan-Cbz/Bphen/LiF-Al, characteristics (*J* and *L vs.* bias voltage) of exciplex system formed using a blend of Xan-Cbz and NPD (1 : 1 by weight); (b) EL of exciplex forming device at different voltages; (c) *J*–*L* characteristics of device, ITO/PEDOT:PSS/CBP/Xan-Cbz/Bphen/LiF-Al; and (d) its EL.

As discussed earlier from the photophysical studies of Xan-Cbz and its blend with NPD in thin film, the peak at ∼465 nm was attributed to the TADF emission of Xan-Cbz and the peak at 525 nm was assigned for exciplex emission. To further verify the exciplex formation under electrical excitation, devices of two different geometries were fabricated. In the first geometry, ITO/PEDOT:PSS/Xan-Cbz + NPD (1 : 1)/Bphen/LiF-Al, having blend of Xan-Cbz and NPD as active emitting layer. The device characteristics shown in Fig. 6(a) shows reasonable light emission (*L* = 2890 Cd m^−2^ @ 50 mA cm^−2^) with a *V*_ON_ = 7.7 V. The EL spectra of this device (Fig. 6(b)) indeed showed a dominant emission at 525 nm consistent with that of the PL observed in thin film. Peak at 525 nm in EL is thus attributed to exciplex formation under electrical excitation. This device showed very little (negligible) effect of varying voltage on EL spectra. In other device CBP was used in place of NPD and the device structure is: ITO/PEDOT:PSS/CBP/Xan-Cbz/Bphen/LiF-Al. Here, CBP was used as an additional HTL. It is worth to mention that blend of Xan-Cbz and CBP (1 : 1) does not show exciplex formation as discussed earlier. Fig. 6(c) shows the device characteristics. Maxima in electroluminescence was obtained at 475 nm which corresponds to emission of Xan-Cbz (Fig. 6(d)). However, these devices seemed to be less efficient than the previous two. Both the current density and light intensity were low from these devices and the *V*_ON_ was quite high (see Fig. 6(c)).

## Experimental

### Materials and methods

Chemicals were purchased from Sigma-Aldrich and used as received. Organic solvents were dried using reported standard procedures. ^1^H and ^13^C NMR spectra were recorded using a Bruker 300 MHz spectrometer. Tetramethylsilane (TMS) in CDCl_3_ was used as an internal reference residual proton; *δ* = 7.26 ppm. Mass spectra were recorded on Bruker MALDI-TOF instrument. CH Instruments 620D electrochemical analyser was used to perform cyclic voltammetry. Anhydrous acetonitrile with tetrabutylammonium hexafluorophosphate solution (0.1 M) as supporting electrolyte at room temperature was taken for measurement. The oxidation potential of Fc/Fc^+^ was used as internal reference. The absorption spectra were recorded using Shimadzu 1800, and emission spectra were recorded on Agilent, Fluorolog-3 and Fluoromax-4 spectrofluorometer. A diode laser-based time-correlated single-photon counting (TCSPC) spectrometer (IBH, U.K.) was used to obtain the time-resolved fluorescence measurements and has been described in detail elsewhere. TADF lifetime decay was observed after a delay of 50 μs on Horiba Fluorolog-3 Spectrofluorometer. Thin films for emission studies were prepared by spin coating using Holmarc HO-TH-05 spin coater at 2000 rpm for 40 s. For OLED device fabrication, PEDOT:PSS was spin coated at 8000 rpm for 40 s. Vacuum deposition was carried out at a base vacuum of 2 × 10^−6^ mbar. ITO-coated substrate (15–25 Ω sq^−1^, Sigma-Aldrich) was cut into 22 × 12 mm^2^. Desired pattern were etched on the ITO substrate using Zn powder and 10% HCl. Substrate was first cleaned with soap solution and distilled water. Then it was sonicated for 10 min each in distilled water and isopropanol. In the last step substrate was cleaned with trichloroethylene vapours. After cleaning, the ITO coated substrate is given UV treatment for 1 h after which the substrates were ready for device fabrication. CBP, NPD, Xan-Cbz, Bphen, LiF and Al where ever required were thermally evaporated in vacuum (base vacuum ∼10^−6^ mbar). For exciplex devices a homogenous solution of NPD + Xan-Cbz (1 mg each) is made in chloroform (0.5 mL) and spin coated at 3000 rpm for 40 s. *J*–*V* measurements of the OLED devices were carried out using a 2400 Keithley sourcemeter at the emitting wavelength. Using lock-in detection the luminance–voltage (*L*–*V*) spectra were also simultaneously recorded. The lock-in signal was converted to light units using the conversion factors, which were estimated using a calibrated light source. Electroluminescence of the devices were measured on a setup consisting of a Bausch and Lomb 350–750 nm monochromator and a Hamamatsu R212 photomultiplier tube as the detector.

## Conclusions

Xanthone–carbazole based organic D-A-D molecule Xan-Cbz was synthesized by substituting carbazole on 2, 7 position of xanthone core. Photophysical studies were performed to establish the TADF in Xan-Cbz. Thin film studies of blends of Xan-Cbz with NPD showed the formation of exciplex. OLED of Xan-Cbz were fabricated using the device architecture as ITO/PEDOT:PSS/NPD/Xan-Cbz/Bphen/LiF-Al. The turn on voltage (*V*_ON_) was ∼6 V and luminous intensity was found to be 1.96 × 10^4^ Cd m^−2^ at a current density of 50 mA cm^−2^. Electroluminescence peaks at 465 and 525 nm were observed which were attributed to emission from neat Xan-Cbz and its exciplex (with NPD) respectively. With increasing voltage the ratio of two peaks changed and colour of device changed from green to greenish blue. Pure TADF and pure exciplex devices were also fabricated and studied.

## Conflicts of interest

There are no conflicts to declare.

## Supplementary Material

RA-009-C9RA08227A-s001
