# Supplementary material for: TADF and exciplex emission in a xanthone–carbazole derivative and tuning of its electroluminescence with applied voltage
Source: RSC Adv. 2019 Dec 4;9(69):40248–54. doi: 10.1039/c9ra08227a (PMC9076223; doi:10.1039/c9ra08227a)
Supplement: RA-009-C9RA08227A-s001 [file RA-009-C9RA08227A-s001.pdf]

Electronic Supplementary Material (ESI) for RSC Advances.
This journal is © The Royal Society of Chemistry 2019

TADF, Exciplex Emission in Xanthone-Carbazole Derivative and Tuning of its Electroluminescence with Applied Voltage

Qamar T. Siddiqui,[†,§] Ankur A. Awasthi,[†] Prabhjyot Bhui,[‡] Pradnya Parab,[‡] Mohammad Muneer,[§] Sangita Bose[‡]* and Neeraj Agarwal[†]*

[†]School of Chemical Sciences, [‡]School of Physical Sciences, UM-DAE, Centre for Excellence in Basic Sciences, University of Mumbai, Santacruz (E), Mumbai, 400098, India.

[§]Department of Chemistry, Aligarh Muslim University, Aligarh, India.

Contents:

Figure S1: ^{1}H (Top) and ^{13}C (Bottom) NMR spectra of **Xan-Cbz** in $CDCl_3$.

Figure S2: Mass spectra of **Xan-Cbz.**

Table S1: Transient lifetime of **Xan-Cbz** in different solvents (in air and nitrogen purged environments) and in thin film.

Figure S3: Normalized absorption spectrum of **Xan-Cbz** in various polarity solvents.

Figure S4: Normalized emission spectra of **Xan-Cbz** in various polarity solvents.

Figure S5: Transient Lifetime measurements (λ_{ex} = 406 nm) of **Xan-Cbz** in DCM under air and nitrogen atmosphere.

Figure S6: Transient Lifetime measurements (λ_{ex} = 406 nm) of **Xan-Cbz** in ACN under air and nitrogen atmosphere.

Figure S7: Decay profile of exciplex of **Xan-Cbz** with **NPD (1:1)** in thin film.

Figure S8: Emission spectrum of **Xan-Cbz** at 77 K in Me-THF.

Figure S9: Cyclic voltammogram of **Xan-Cbz** in ACN.

Figure S10: Current efficiencies in Cd/A as a function of current density for the devices reported in the manuscript.

Figure S11: Normalized EL for one of the device with the geometry ITO/PEDOT:PSS/NPD/**Xan-Cbz**/Bphen/LiF/Al which was stored and measured in vacuum . The red curve is the normalized EL spectra (normalized at λ = 525 nm) taken for the device immediately after fabrication at a voltage of 15 V while the blue curve is for the same device recorded after 30 days at the same bias voltage. The EL spectra looks the same indicating colour stability. The intensity at λ = 468 nm has decreased slightly indicating a small degradation of the device with time.

Figure S12: Structure of Xan-Cbz and reported molecules having similar luminous intensity.

Table S2: Comparision of luminous intensity of Xan-Cbz with similar reported molecules.

Scheme S1: Reaction steps for synthesis of **Xan-Cbz** from xanthone and carbazole.

Synthesis and Purification: Synthesis and Chemical methods for purification of **Xan-Cbz.**

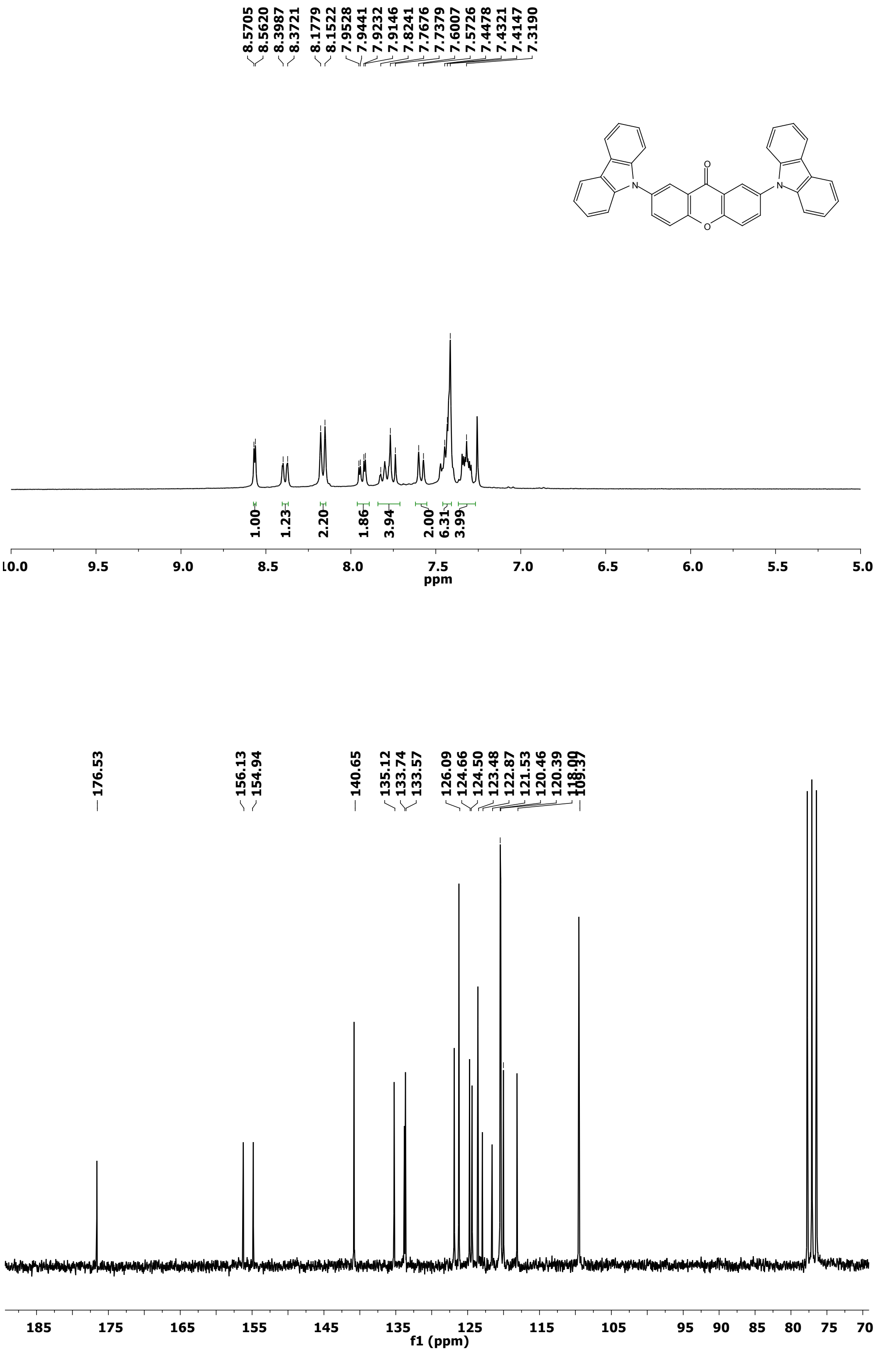

Figure S1: ^{1}H (Top) and ^{13}C (Bottom) NMR spectra of **Xan-Cbz** in $CDCl_3$.

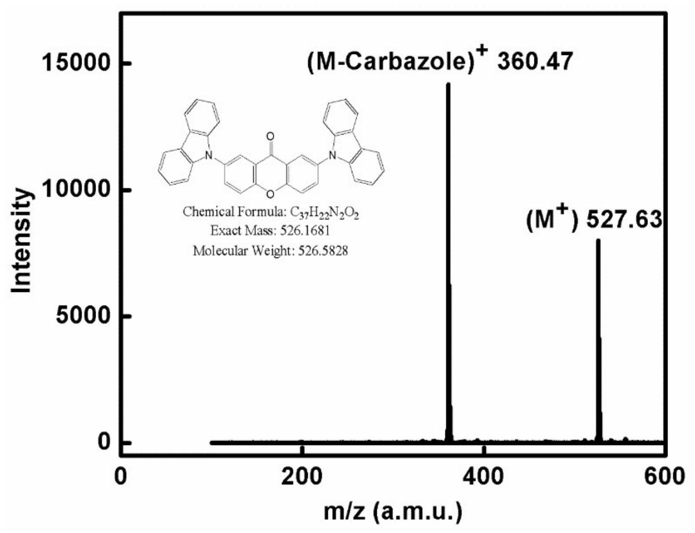

Figure S2: Mass spectra of **Xan-Cbz**

Table S1: Transient lifetime of **Xan-Cbz** in different solvents (in air and nitrogen purged environments) and in thin film

Solvent	In Oxygen			In Nitrogen		
	τ_1/ns	τ_2/ns	τ_3/ns	τ_1/ns	τ_2/ns	τ_3/ns
THF	0.84	11.21	-	1.66	16.40	-
DCM	1.99	18.61	-	2.01	24.42	-
ACN	0.29	2.32	9.30	0.48	3.65	12.57

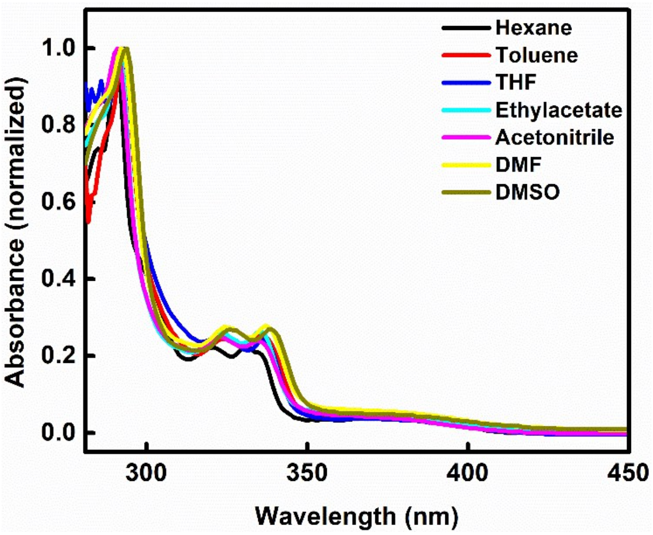

Figure S3: Normalized absorption spectrum of **Xan-Cbz** in various polarity solvents.

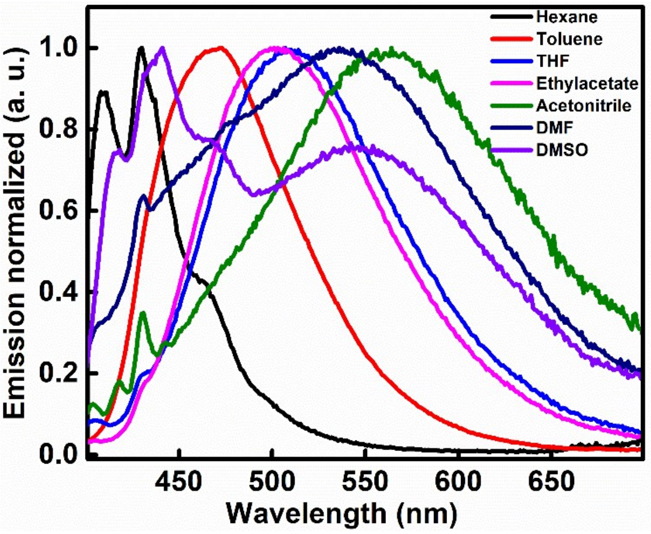

Figure S4: Normalized emission spectra of **Xan-Cbz** in various polarity solvents.

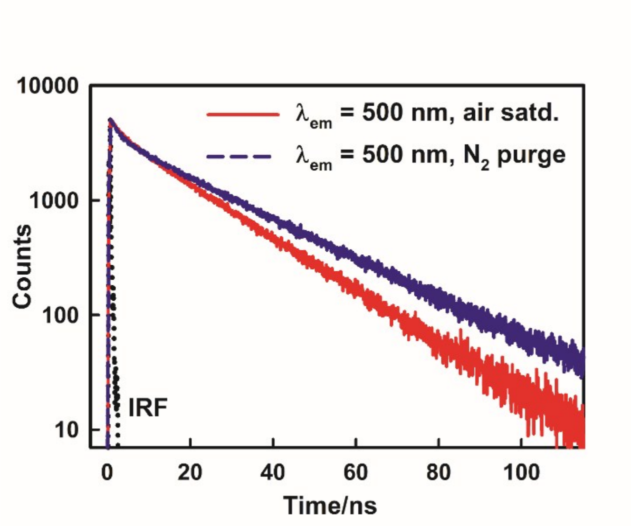

Figure S5: Transient Lifetime measurements (λ_{ex} = 406 nm) of **Xan-Cbz** in DCM under air and nitrogen atmosphere.

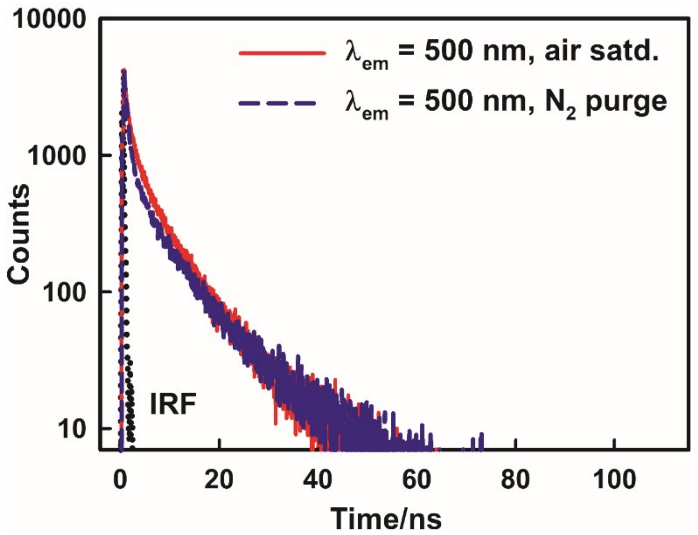

Figure S6: Transient Lifetime measurements (λ_{ex} = 406 nm) of **Xan-Cbz** in ACN under air and nitrogen atmosphere.

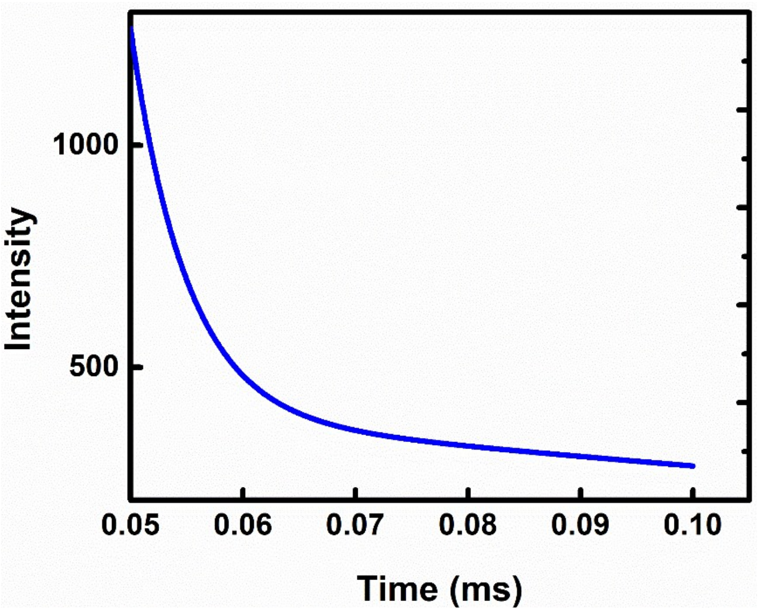

Figure S7: Decay profile of exciplex of **Xan-Cbz** with **NPD (1:1)** in thin film.

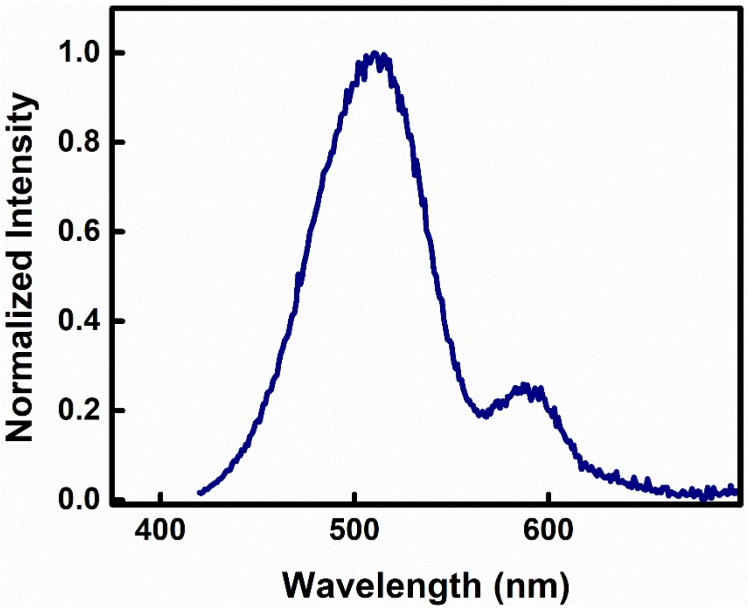

Figure S8: Emission spectrum of **Xan-Cbz** at 77 K in Me-THF.

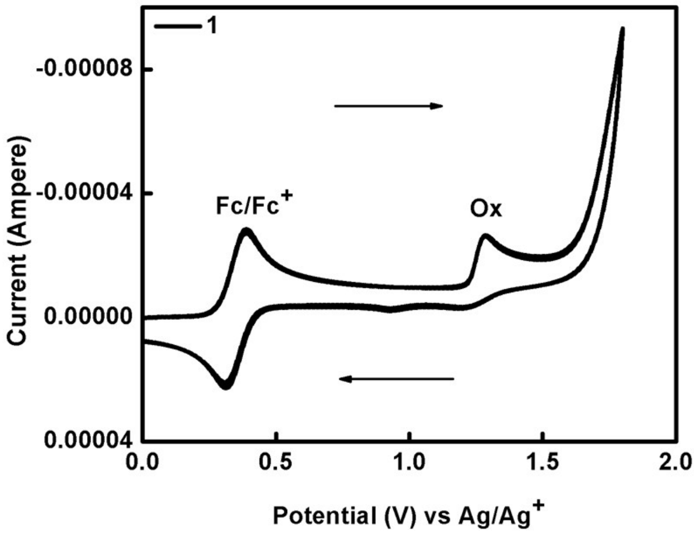

Figure S9: Cyclic voltammogram of **Xan-Cbz** in ACN.

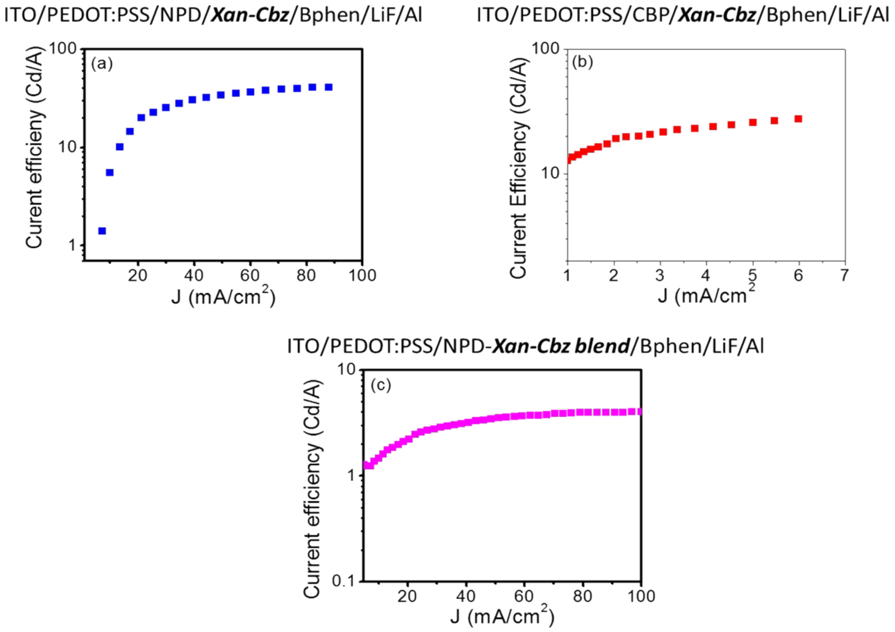

Figure S10: Current efficiencies in Cd/A as a function of current density for the devices reported in the manuscript.

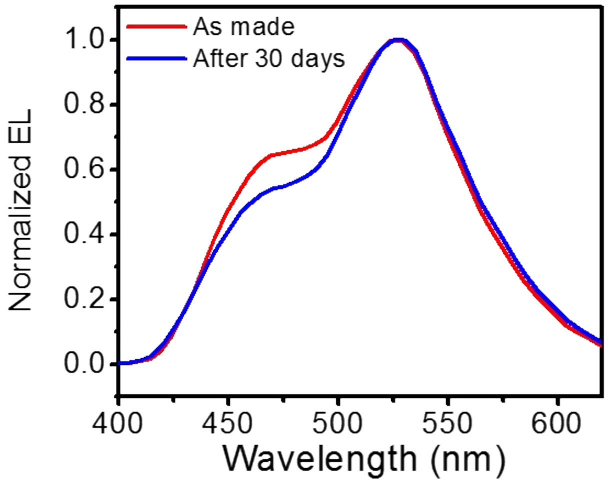

Figure S11: Normalized EL for one of the device with the geometry ITO/PEDOT:PSS/NPD/**Xan-Cbz**/Bphen/LiF/Al which was stored and measured in vacuum . The red curve is the normalized EL spectra (normalized at λ = 525 nm) taken for the device immediately after fabrication at a voltage of 15 V while the blue curve is for the same device recorded after 30 days at the same bias voltage. The EL spectra looks the same indicating colour stability. The intensity at λ = 468 nm has decreased slightly indicating a small degradation of the device with time.

Table S2: Comparision of luminous intensity of Xan-Cbz with similar reported molecules.

Compound	Luminous intensity (Cd/m^2)	Current density (mA/cm^2)
**Xan-Cbz**	1.9×10^4	50
**1**	1.0×10^3	60
**2**	1.0×10^3	10
**3**	1.7×10^4	25
**4**	9.8×10^3	10

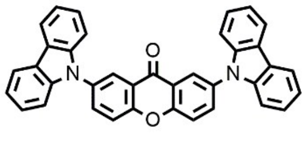

Xan-Cbz

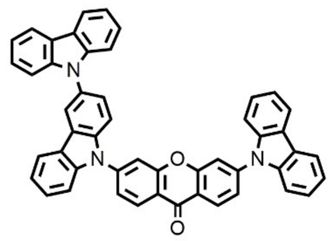

1

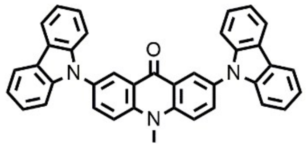

2

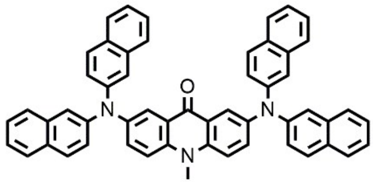

3

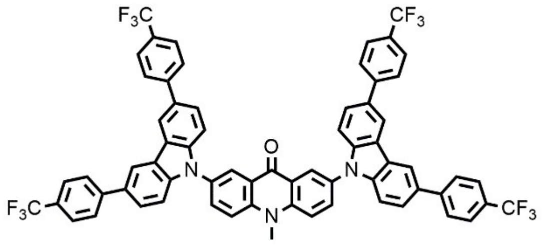

4

Figure S12: Structure of Xan-Cbz and reported molecules having similar luminous intensity.[1, 2,3]

Synthesis

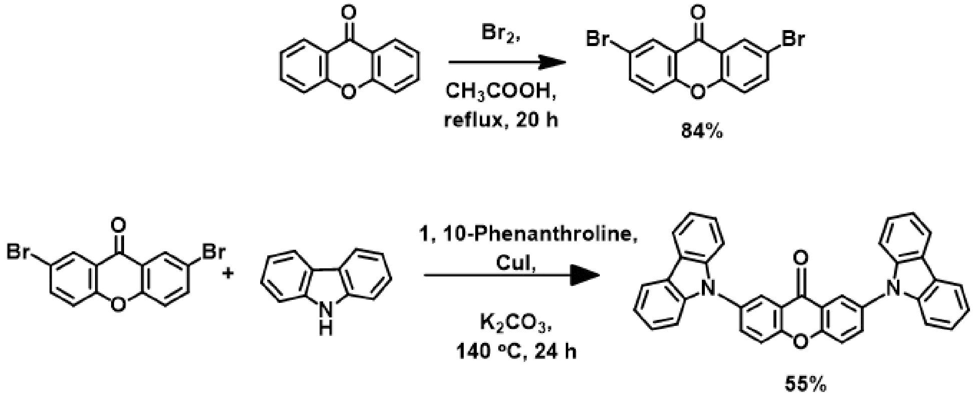

Scheme S1: Reaction steps for synthesis of **Xan-Cbz** from xanthone and carbazole.

2,7-di(9H-carbazol-9-yl)-9H-xanthen-9-one, Xan-Cbz A mixture of 2,7-dibromo-9H-xanthen-9-one (200 mg, 0.564 mmol), Carbazole (282 mg, 1.692 mmol), Copper(I)Iodide (320 mg, 1.692 mmol), potassium carbonate (390 mg, 2.8 mmol) and 1, 10-phenanthroline (200 mg, 1.128 mmol) was dissolved in 5 mL of dry DMF. The reaction is refluxed for 24 hours under nitrogen. The resulting mixture is cooled, poured in water and extracted with DCM. The compound is purified with column chromatography using 30% ethyl acetate in hexane as eluent.

The ^{1}H NMR spectra of the above purified product showed impurities of carbazole. The product and carbazole, both moved together in column as they have similar R_f values. To remove the carbazole impurity the product was reacted in DMSO with methyl iodide in presence of 50% NaOH at 80 ^{0}C for 6 hours. The left over carbazole is methylated to form N-methyl carbazole whose R_f value is different from **Xan-Cbz**. The final product is then extracted with DCM and water, purified by column chromatography and further purified by recrystallization to get pure product. (Yield: 55%)

^{1}H NMR (CDCl3, 300 MHz, δ ppm): 8.56 (s, 1H), 8.38 (d, 1H, J = 7.9 Hz); 8.16 (d, 2H, J = 7.7 Hz); 7.93(d, 2H, J = 8.8 Hz); 7.78 (m, 4H); 7.58 (d, 2H, J = 8.4 Hz); 7.43 (m, 6H); 7.31 (m, 4H); ^{13}C NMR (CDCl3, 75 MHz, δ ppm): 176.53; 156.13; 154.94; 140.65; 135.12; 133.74; 133.57; 126.06; 124.66; 124.50; 123.48; 122.87; 121.53; 120.46; 120.39; 118.00; 109.37; MALDI-TOF: m/z [M]+ calcd. $C_{37}H_{22}N_2O_2$, 526.58; found: 527.63.

References:

(1) Miwa, T.; Kubo, S.; Shizu, K.; Komino, T.; Adachi, C.; Kaji, H., Blue Organic Light-emitting Diodes Realizing External Quantum Efficiency Over 25% Using Thermally Activated Delayed Fluorescence Emitters. *Sci. Rep.* **2017,** *7*, No. 284.
(2) Siddiqui, Q. T.; Awasthi, A. A.; Bhui, P.; Muneer, M.; Chandrakumar, K. R. S.; Bose, S.; Agarwal, N., Thermally Activated Delayed Fluorescence (Green) in Undoped Film and Exciplex Emission (Blue) in Acridone–Carbazole Derivatives for OLEDs. *J. Phys. Chem. C* **2019,** *123*, 1003-1014.
(3) Awasthi, A. A.; Gupta, N.; Siddiqui, Q. T.; Parab, P.; Palit, D. K.; Bose, S.; Agarwal, N., Synthesis of Acridone-amine Derivative and its Thermally Activated Delayed Fluorescence Properties for Application in OLEDs. (**Accepted in J. Chem. Sci.**)
